# The Degree of Anæsthesia Which Should Be Induced Prior to Surgical Operations

**Published:** 1897-06

**Authors:** 


### SELECTIONS.

### The Degree of Anæsthesia Which Should Be Induced Prior to Surgical Operations.

Within the last few years we have called attention more than once, in the editorial columns of the *Therapeutic Gazette* to the importance of producing complete anæsthesia before beginning an operation, particularly when chloroform is the anæsthetic employed. In one of these editorials we emphasized our belief that fright has a very marked influence in increasing the danger which is ever present when chloroform is administered; and in another we pointed out that a possible cause of the large number of fatalities during the early part of the administration of chloroform might be traced to the fact that, combined with fright, there was sent to the heart a powerful reflex through the sensory and pneumogastric nerves as soon as the knife touched the skin of the patient or the surgical operation was begun. This is particularly apt to be the case under chloroform, because it would seem probable that early in the administration of this drug, while there may be some interference with consciousness and some benumbing of the ordinary sense of pain, there is not an abolition of tactile sense, but, on the other hand, rather a condition of excessive irritability of the nerves involved in this sense. Further experience has intensified our belief that our earlier views were correct, and the writings of leading surgeons and anæsthetists the world over seem to point more and more to the correctness of this opinion. As an instance of this, we may quote the statements made by Dr. Hewitt, of London, in regard to this question. He points out that one chloroformist will work with light anesthesia, another with moderately deep anesthesia, and another with very deep anesthesia, but the success of Syme in administering chloroform appears to have been principally due to the almost invariable maintenance of a profound narcosis. He also points out what the writer of this editorial has also pointed out in previous contributions to medical literature, that the very development of a profound narcosis tends to render the respiration regular and unhampered; whereas, the administration of insufficient quantities of chloroform causes the respiration to be so irregular and uneven that it is impossible for the anesthetist, during the course of a prolonged operation, to make any accurate estimation of the quantity of chloroform which the patient is receiving. In other words, a part of the skill of an anesthetist lies in his ability to give a sufficient quantity to induce deep anesthesia without giving enough to produce a condition of danger.

In many instances the surgeon is inclined to regard the early delays in the administration of an anesthetic as being a useless waste of time; and we have seldom administered an anesthetic for a surgeon, or watched its administration for one, without being impressed with the fact that his tendency is continually to hurry the anesthetist, with the result that the drug is often at first pushed more freely than the anesthetist himself believes to be proper. In our opinion, the most delicate stage in the administration of an anesthetic is during the period when the patient is passing from complete consciousness into unconsciousness, and, if the patient is well chloroformed, far less skill and knowledge is required to maintain the anesthesia in a condition of safety than was necessary earlier in the process.—*Therapeutic Gazette.*

